# Assessment of Recommendations Provided to Athletes Regarding Sleep Education by GPT-4o and Google Gemini: Comparative Evaluation Study

**DOI:** 10.2196/71358

**Published:** 2025-07-08

**Authors:** Lukas Masur, Matthew Driller, Haresh Suppiah, Manuel Matzka, Billy Sperlich, Peter Düking

**Affiliations:** 1 Department of Sports Science and Movement Pedagogy, Technische Universität Braunschweig, Pockelsstraße 11, Braunschweig, 38106, Germany, 49 531 391 3432; 2 School of Allied Health, Human Services, and Sport, La Trobe University, Melbourne, Australia; 3 Department of Sports Science, Integrative and Experimental Exercise Science & Training, University of Würzburg, Würzburg, Germany

**Keywords:** individualization, personalization, artificial intelligence, education, recovery, monitoring

## Abstract

**Background:**

Inadequate sleep is prevalent among athletes, affecting adaptation to training and performance. While education on factors influencing sleep can improve sleep behaviors, large language models (LLMs) may offer a scalable approach to provide sleep education to athletes.

**Objective:**

This study aims (1) to investigate the quality of sleep recommendations generated by publicly available LLMs, as evaluated by experienced raters, and (2) to determine whether evaluation results vary with information input granularity.

**Methods:**

Two prompts with differing information input granularity (low and high) were created for 2 use cases and inserted into ChatGPT-4o (GPT-4o) and Google Gemini, resulting in 8 different recommendations. Experienced raters (n=13) evaluated the recommendations on a 1‐5 Likert scale, based on 10 sleep criteria derived from recent literature. A Friedman test with Bonferroni correction was performed to test for significant differences in all rated items between the training plans. Significance level was set to *P*<.05. Fleiss κ was calculated to assess interrater reliability.

**Results:**

The overall interrater reliability using Fleiss κ indicated a fair agreement of 0.280 (range between 0.183 and 0.296). The highest summary rating was achieved by GPT-4o using high input information granularity, with 8 ratings >3 (tendency toward good), 3 ratings equal to 3 (neutral), and 2 ratings <3 (tendency toward bad). GPT-4o outperformed Google Gemini in 9 of 10 criteria (*P*<.001 to *P*=.04). Recommendations generated with high input granularity received significantly higher ratings than those with low granularity across both LLMs and use cases (*P*<.001 to *P*=.049). High input granularity leads to significantly higher ratings in items pertaining to the used scientific sources (*P*<.001), irrespective of the analyzed LLM.

**Conclusions:**

Both LLMs exhibit limitations, neglecting vital criteria of sleep education. Sleep recommendations by GPT-4o and Google Gemini were evaluated as suboptimal, with GPT-4o achieving higher overall ratings. However, both LLMs demonstrated improved recommendations with higher information input granularity, emphasizing the need for specificity and a thorough review of outputs to securely implement artificial intelligence technologies into sleep education.

## Introduction

Sleep is essential for the health and well-being of individuals across all age groups, as it supports cognitive function, mood, mental health, as well as cardiovascular, cerebrovascular, and metabolic health [1,2]. Short-term sleep deprivation, chronic sleep restriction, circadian misalignment, and untreated sleep disorders can significantly harm physical health, mental health, and mood [1,3,4].

One population frequently experiencing inadequate sleep or poor sleep quality is athletes [5-7]. Reasons for poor sleep may stem from multiple sport and nonsport factors including high training loads, long-haul travel, early morning training, family commitments, lifestyle choices including diet, or work or study commitments [6]. While it is beyond the scope of this paper to dive into the effects of inadequate sleep in detail, we refer the reader to existing papers on this topic [6,8]. Briefly, athletes’ sleep is considered a primary mechanism facilitating both psychological and physiological recovery [9,10]. Poor sleep may be detrimental for athletes, with negative impacts on mental well-being, cognition, learning and memory consolidation, growth and repair of cells, glucose metabolism, and immune responses (eg, the resistance to respiratory infection) [6,11,12].

To improve aspects of sleep, a first step is to educate athletes and staff on the negative effects of poor sleep and factors affecting sleep [6], and there is evidence that sleep education improves the sleep behavior of team sport athletes [13]. Such sleep education might include (1) education of athletes on potential factors negatively impacting sleep [14] and (2) education on reducing the impact of factors negatively impacting sleep.

It was reported that even in elite athlete cohorts, there is a lack of sleep knowledge [15], and consequently, there is a need for sleep education. For this, artificial intelligence and, more specifically, publicly available large language models (LLMs) might offer a scalable solution. By simulating human-like conversations, LLMs leverage deep learning to process information and generate nuanced responses. LLMs such as ChatGPT (OpenAI) are rapidly gaining popularity among the general population [16] as well as in various scientific domains such as medical research and education [17-20], health care [21-23], or nutrition [24,25]. Thereby, it is likely that individuals turn to LLMs to receive responses to questions they face, for example, regarding sleep. However, it is currently unknown if recommendations regarding sleep generated by publicly available LLMs are appropriate and in line with recent scientific evidence and thus suitable for a specific athlete. Here, we aim (1) to investigate sleep recommendations provided to athletes generated by different publicly available LLMs as evaluated by experienced raters and (2) to investigate if sleep recommendations differ depending on the provided information by the user.

## Methods

### General Design

**Figure 1.**
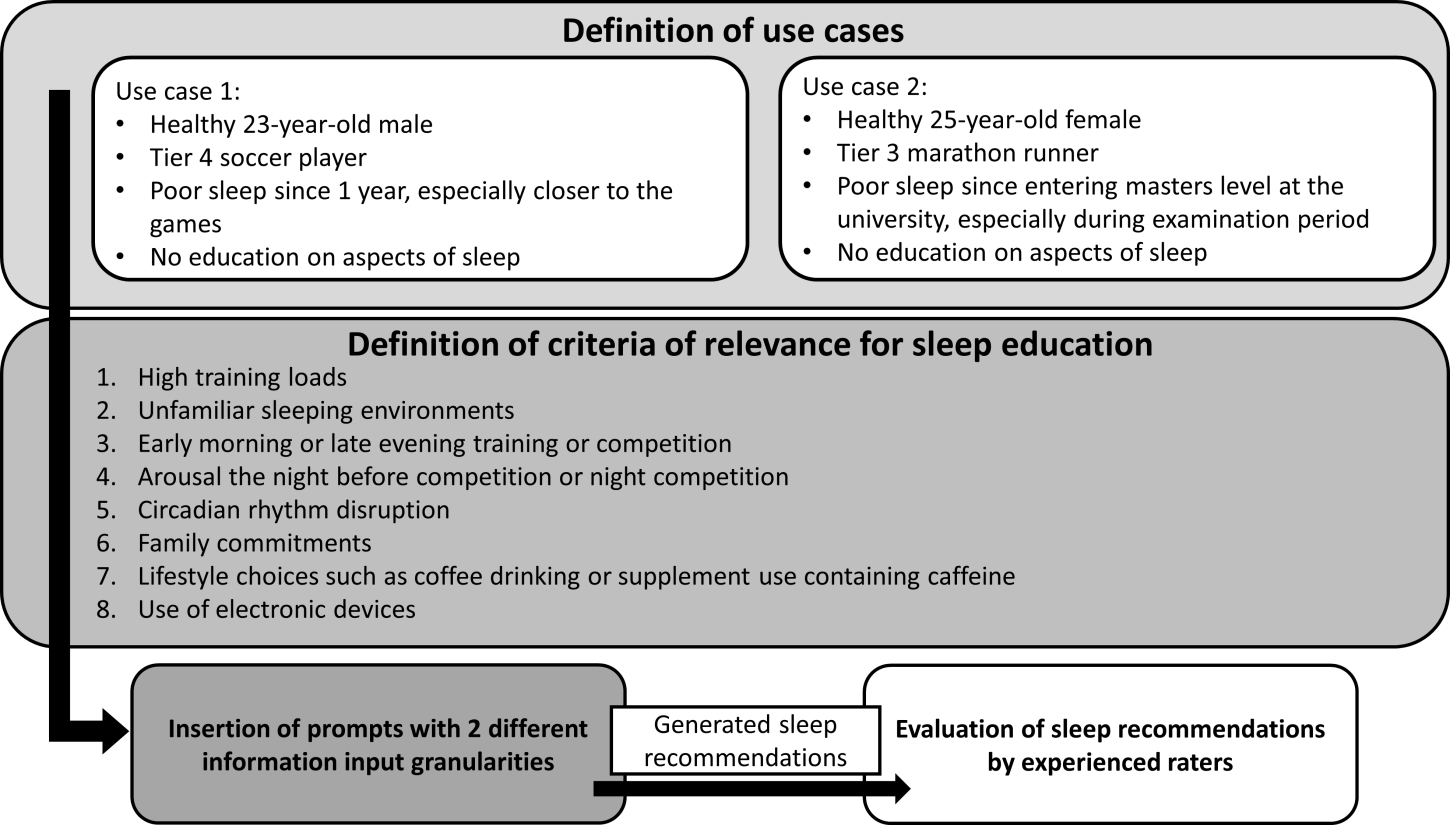
Experimental workflow of the study.

To evaluate sleep recommendations provided by LLMs, we followed methodologies of similar papers in the medical field [26-29] or in the exercise science literature [30-33] and adjusted these methodologies to the aim of our research. Figure 1 depicts the experimental workflow of the study.

For this, we (1) define a specific use case, (2) define criteria of relevance for sleep education in this use case, (3) define information input into publicly available LLMs, and (4) involve experienced raters in the topic of sleep within the athletic population to rate outcomes of LLM-generated responses.

### Ethical Considerations

The ethics committee of the Faculty of Exercise Science and Training at the University of Würzburg approved the study (reference: EV2025/5-0606). Raters were informed about procedures and gave their consent to participate in the study. No compensation was given to the raters. After receiving the ratings, these were deidentified.

### Definition of 2 Use Cases

#### Use Case 1: Male Tier 4 Soccer Player

For use case 1, we define a healthy, 23-year-old male tier 4, elite soccer player [34] who trains 5 times a week and has 1‐2 competitive games per week at a national level. The soccer player experiences poor sleep for approximately a year, and sleep disturbance is closer to important games. The individual in our use case has no formal education on, for example, factors affecting sleep or on the effectiveness of countermeasures to improve sleep and no access to experienced and educated personnel who could educate him on sleep. We define that the major reasons for impaired sleep are high training loads, arousal the night before competition, and unfamiliar sleeping environments in the case of away games.

#### Use Case 2: Female Tier 3 Marathon Runner

For use case 2, we define a healthy, 25-year-old female tier 3, highly trained or national-level marathon runner [34] who runs around 100 km per week following a pyramidal training intensity distribution [35]. The female runner competes at the national level. In addition to her running training, the runner is enrolled at a master level at university.

The female runner experiences poor sleep since entering master level at university (approximately 6 months ago), and sleep is compromised especially during examination periods, which also affects running training and performance. The individual in our use case has no formal education on, for example, factors affecting sleep or on the effectiveness of countermeasures to improve sleep and no access to experienced and educated personnel who could educate her on sleep. We define that the major reasons for impaired sleep are training loads and disturbances stemming from university obligations.

In line with the aims of this research, we challenge the publicly available LLMs (1) to identify the reasons for sleep disturbances and (2) to give evidence-based guidance on how to reduce the impact of factors negatively impacting sleep.

### Criteria of Relevance for Sleep Education

There are many sport and nonsport factors that impact the sleep of athletes. However, factors influencing sleep that are most commonly mentioned in the literature include high training loads [6,14], unfamiliar sleeping environments [6], early morning or late evening training or competition [6,14], arousal the night before competition or the night after competition [6,14], circadian rhythm disruption (eg, due to long-haul travel) [6,14], family commitments [6], lifestyle choices such as coffee drinking or supplement use containing caffeine [6,14], and use of electronic devices (eg, smartphone use) [14].

### Prompts Inserted Into the LLMs

Given the chatbot nature of LLMs, we assume that the input provided by individuals seeking sleep education will vary, like any other conversation. Depending on factors such as previous knowledge about sleep or personal experiences, we assume that some individuals may provide minimal information, while others may be more detailed. To accommodate this diversity in the input information, we developed 2 distinct input information scenarios for both our use cases. Scenario 1 resembles an individual who inserts little to no information and only asks superficial check-backs once a response is given by LLMs. Scenario 2 resembles an individual who inserts more information and asks more detailed check-backs once a response is given by LLMs. The complete conversations with LLMs are available in MultimediaAppendices 1  2. Prompts of the scenarios were designed by the authors who are frequently in conversations with athletes on aspects of sleep.

For use case 1, the initial prompts were as follows:

Scenario 1:

I feel tired in the morning and after waking up. I do not know why. This is especially worse close to soccer games. Can you give me advice on how to improve my sleep?

Scenario 2:

I am a 23 year old male highly trained/national level soccer player. I train 5 times a week and plus a game per week. I feel tired due to poor sleep at night since approximately a year. Especially the night before a game I sleep poorly. Can you give me advice on factors which might affect my sleep and how I can reduce the impact of these factors? Use only scientific literature and specifically, for each advice, state this literature and provide a reference list at the end.

For use case 2, the initial prompts were as follows:

Scenario 1:

I feel tired in the morning and after waking up. I do not know why. Maybe it is due to my running training or due to entering master level at university. Can you give me advice on how to improve my sleep?

Scenario 2:

I am a 25 year old female tier 3, highly trained/national level marathon runner player and I run approx. 100km per week with a pyramidal intensity distribution. I feel tired in the morning since approximately entering my master course at university. Can you give me advice on factors which might affect my sleep and how I can reduce the impact of these factors? Use only scientific literature and specifically, for each advice, state this literature and provide a reference list at the end.

Here, we used GPT-4o and Google Gemini Advanced without any use of plug-ins, as both are publicly available and thereby can be used by individuals who seek sleep education. Prompts were inserted on May 15, 2024.

### Raters

We reached out to well-educated and experienced raters on sleep and athletes to assess the provided recommendations on the outlined aspects relevant for sleep education on a 1 to 5 Likert scale. We included a total of 13 experienced raters (age span: 28‐42 years; n=6 with a PhD, n=7 with a master degree in human physiology or sleep science) working for an average of 9 (SD 7) years with athletes on matters of sleep and indicating a mean sleep research experience of 4 (SD 6) years participated in this study.

### Statistical Analysis

We calculated descriptive statistics for the Likert scores on all rated items for each question. To test for significant differences in all rated items between the training plans, a Friedman test with Bonferroni correction was performed. Significance level was set to *P*<.05. Fleiss κ was calculated to assess interrater reliability [36]. Interpretation of Fleiss κ results was conducted according to the classification by Landis and Koch [37]. Fleiss κ values were interpreted as follows: a value of 0.00-0.20 as “slight,” 0.21-0.40 as “fair,” 0.41-0.60 as “moderate,” 0.61-0.80 as “substantial,” and >0.80 as “almost perfect” [37]. All statistical analyses were performed in SPSS (version 28; IBM Corp).

## Results

### Overview

Table 1 represents Fleiss κ values for the different LLMs and use cases. The analysis of Fleiss κ indicated a fair agreement of the overall interrater reliability (0.280), while results ranged between 0.183 and 0.296 (Table 1).

Descriptive statistics of the evaluated sleep recommendations are presented in Table 2.

**Table 1.** Fleiss κ results.

Large language model, use case, and scenario		Fleiss κ
Google Gemini		
	Use case 1—scenario 1 (Gem_C1-S1)	0.270
	Use case 1—scenario 2 (Gem_C1-S2)	0.198
	Use case 2—scenario 1 (Gem_C2-S1)	0.296
	Use case 2—scenario 2 (Gem_C2-S2)	0.183
GPT-4o		
	Use case 1—scenario 1 (GPT_C1-S1)	0.258
	Use case 1—scenario 2 (GPT_C1-S2)	0.264
	Use case 2—scenario 1 (GPT_C2-S1)	0.256
	Use case 2—scenario 2 (GPT_C2-S2)	0.229

**Table 2.** Descriptive analysis of Likert-scale ratings^a^.

Relevant aspects when deploying sleep recommendations		Gem_C1-S1^b^	GPT_C1-S1^c^	Gem_C1-S2^d^	GPT_C1-S2^e^	Gem_C2-S1^f^	GPT_C2-S1^g^	Gem_C2-S2^h^	GPT_C2-S2^i^
General aspects, median (IQR)									
	Overall training plan	3 (3-3)	3 (3-4)	3 (3-4)	4 (4-4)	3 (3-4)	3 (3-4)	4 (3-4)	4 (3.75-4)
	Training load	0 (0-3)	0 (0-0)	3 (0-4)	4 (3-4)	3 (2-4)	4 (3-4)	4 (3-4)	4 (4-5)
	Unfamiliar sleeping environments	0 (0-3)	2 (0-3)	4 (3-4)	3 (0-4)	3 (0-3)	3 (0-4)	3 (0-4)	3 (0-4)
	Early morning or late evening training or competition	3 (0-4)	3 (3-3)	4 (4-4)	4 (4-4)	4 (3-4)	3 (0-4)	0 (0-4)	4 (3-4)
	Arousal the night before competition or training	0 (0-3)	3 (3-4)	4 (3-4)	3 (3-4)	0 (0-0)	0 (0-0)	0 (0-0)	0 (0-4)
	Circadian rhythm disruptions or consistency of sleep schedule	3 (3-4)	4 (3-4)	4 (4-4)	4 (3-5)	4 (3-4)	3 (3-4)	4 (3-4)	3 (3-4)
	Family commitments	0 (0-0)	0 (0-0)	0 (0-0)	0 (0-0)	0 (0-0)	0 (0-0)	0 (0-0)	0 (0-0)
	Lifestyle choices	3 (0-3)	3 (0-4)	2 (0-3)	0 (0-3)	3 (2-4)	3.5 (2.25-4)	3 (0-3)	3 (3-3)
	Use of electronic devices	3 (3-3.25)	4 (3-5)	0 (0-0)	4 (4-5)	3 (0-4)	4 (3-4)	4 (3-4)	4 (3-5)
	Nutrition	3 (3-3)	4 (3-4)	4 (3-4)	3 (3-5)	4 (3-4)	3 (3-4)	4 (3-4)	4 (3-5)
Summary rating, n (%)									
	<3	4 (40)	3 (30)	3 (30)	2 (20)	2 (20)	2 (20)	3 (30)	2 (20)
	3	6 (60)	4 (40)	2 (20)	3 (30)	5 (50)	5 (50)	2(20)	3 (30)
	>3	0 (0)	3 (30)	5 (50)	5 (50)	3 (30)	3 (30)	5 (50)	5 (50)
Scientific sources, median (IQR)									
	Is the real and existing literature stated (no “fake citations”)?	0 (0-0)	0 (0-0)	4 (2-4)	5 (5-5)	0 (0-0)	0 (0-0)	4 (3-4)	5 (5-5)
	Appropriateness of provided scientific evidence	0 (0-0)	0 (0-0)	4 (3-4)	4 (4-4)	0 (0-0)	0 (0-0)	4 (4-5)	4 (4-5)
	Quality of provided scientific evidence in this specific context	0 (0-0)	0 (0-0)	4 (3-4)	4 (4-4)	0 (0-0)	0 (0-0)	4 (3-4)	4 (4-4)
Summary rating, n (%)									
	<3	3 (100)	3 (100)	0 (0)	0 (0)	3 (100)	3 (100)	0 (0)	0 (0)
	3	0 (0)	0 (0)	0 (0)	1 (33.3)	0 (0)	0 (0)	0 (0)	0 (0)
	>3	0 (0)	0 (0)	3 (100)	2 (66.6)	0 (0)	0 (0)	3 (0)	3 (100)

a Results range from 1=bad to 5=good with 0=not applicable.

b Gem_C1-S1: Google Gemini, use case 1—scenario 1.

c GPT_C1-S1: ChatGPT, use case 1—scenario 1.

d Gem_C1-S2: Google Gemini, use case 1—scenario 2.

e GPT_C1-S2: ChatGPT, use case 1—scenario 2.

f Gem_C2-S1: Google Gemini, use case 2—scenario 1.

g GPT_C2-S1: ChatGPT, use case 2—scenario 1.

h Gem_C2-S2: Google Gemini, use case 2—scenario 2.

i GPT_C2-S2: ChatGPT, use case 2—scenario 2.

### Differences Regarding Input Information Granularity and Between Google Gemini and GPT-4o

Results for significance testing regarding different input information granularities and differences between Google Gemini and GPT-4o are presented in Table 3.

Significance testing of the comparison between identical prompts across different LLMs shows that GPT-4o attained significantly higher Likert-scale scores in 9 of 10 criteria of relevance for sleep education (*P*<.001 to *P*=.045). Higher input information granularity, independent of the LLM used, exhibits significantly higher Likert-scale ratings in 28 of 52 criteria items of relevance for sleep education (*P*<.001 to *P*=.049).

In this paper, we compare the output of different LLMs when the same information was inserted. We do not show comparisons of different LLMs and different information input (eg, Gem_C1-S1 vs GPT_C1-S2) but provide these to the interested reader in Multimedia Appendix 3.

**Table 3.** Results of the significance testing comparing training plans: between Google Gemini and GPT-4o for the same input information granularity and within Google Gemini or GPT-4o for different input information granularity.

Relevant aspects when deploying sleep recommendations		Significance testing (*P* value) (Google Gemini versus GPT-4o; same prompt, different LLM)^a^				Significance testing (*P* value) (input information granularity; different prompt, same LLM)			
		Gem_C1-S1^b^ versus GPT_ C1-S1^c^	Gem_C1-S2^d^ versus GPT_ C1-S2^e^	Gem_C2-S1^f^ versus GPT_ C2-S1^g^	Gem_C2-S2^h^ versus GPT_ C2-S2^i^	Gem_C1-S1 versus Gem_C1-S2	GPT_C1-S1 versus GPT_C1-S2	Gem_C2-S1 versus Gem_C2-S2	GPT_C2-S1 versus GPT_C2-S2
General aspects									
	Overall training plan	.92	*.02*	.16	.07	.47	*.003*	.38	*.005*
	Training load	.19	.21	.06	.21	*.003*	*<.001*	*.005*	*.03*
	Unfamiliar sleeping environments	.63	.21	.76	.72	*.006*	.28	.15	.37
	Early morning or late evening training or competition	.88	.88	.08	*.004*	*.01*	*.01*	*.009*	*.04*
	Arousal the night before competition or training	*.005*	.85	.11	*.02*	*.001*	.54	.14	.35
	Circadian rhythm disruptions or consistency of sleep schedule	.16	.96	.43	.96	*.01*	.25	.71	.71
	Family commitments	>.99	*.049*	>.99	>.99	*.049*	>.99	>.99	>.99
	Lifestyle choices	.74	.96	.74	.55	.37	.21	.28	.41
	Use of electronic devices	.07	*<.001*	.27	.38	*<.001*	.22	.48	.64
	Nutrition	*.03*	.70	.96	.28	*.046*	.83	.74	.14
Scientific sources									
	Is real and existing literature stated (no “fake citations”)?	.62	*<.001*	.22	*<.001*	*<.001*	*<.001*	*<.001*	*<.001*
	Appropriateness of provided scientific evidence	.92	.52	.17	.40	*<.001*	*<.001*	*<.001*	*<.001*
	Quality of provided scientific evidence in this specific context	*.04*	.72	.25	.08	*<.001*	*<.001*	*<.001*	*<.001*

a LLM: large language model.

b Gem_C1-S1: Google Gemini, use case 1—scenario 1.

c GPT_C1-S1: ChatGPT, use case 1—scenario 1.

d Gem_C1-S2: Google Gemini, use case 1—scenario 2.

e GPT_C1-S2: ChatGPT, use case 1—scenario 2.

f Gem_C2-S1: Google Gemini, use case 2—scenario 1.

g GPT_C2-S1: ChatGPT, use case 2—scenario 1.

h Gem_C2-S2: Google Gemini, use case 2—scenario 2.

i GPT_C2-S2: ChatGPT, use case 2—scenario 2.

## Discussion

### Principal Findings

We aimed (1) to investigate the quality of sleep recommendations provided to athletes generated by different publicly available LLMs as evaluated by experienced raters and (2) to investigate if the quality of sleep recommendations differs depending on the provided information by the user.

Our main results are as follows:

- The highest Likert-scale rating was achieved by GPT-4o using the prompt with high input granularity (use case 2, scenario 2) with 8 ratings >3 (tendency toward good), 3 ratings equal 3 (neutral), and 2 ratings <3 (tendency toward bad) on a 1‐5 Likert Scale. This indicates that even the highest-ranked recommendations provided by the herein investigated LLMs are not optimal.
- Sleep recommendations by GPT-4o received higher Likert-scale ratings compared to those by Google Gemini (9 of 10 significant differences, with *P*<.001 to *P*=.04). This suggests a tendency that GPT-4o outperforms Google Gemini in the investigated sleep deficiency scenarios.
- Quality of sleep recommendations enhances with higher input information granularity (significantly higher Likert-scale ratings in 28 of 52 criteria items of relevance for sleep education; *P*<.001 to *P*=.049); however, some criteria of relevance for sleep education were partly or completely omitted, irrespective of the input information granularity.

### Ratings of Generated Recommendations Regarding Sleep

Our results indicate that sleep recommendations of publicly available LLMs are not rated optimally, even when inserting a prompt with a high input information granularity. Although prompting GPT-4o with high input information granularity (user case 2, scenario 2) gained the highest Likert-scale ratings by the experienced raters (n=8 >3 Likert-scale rating), the summarized ratings demonstrate that the sleep recommendations exhibit deficiencies (n=3 ratings equal 3 [neutral]; n=2 ratings <3 [tendency toward bad]).

The insufficiency in providing optimal recommendations aligns with previous studies assessing LLMs in other research fields. For example, a systematic review and meta-analysis on ChatGPT’s performance in answering medical questions showed that ChatGPT has an overall accuracy of 56%, suggesting potential but inadequacy for independent clinical decision-making [38]. Research in the field of nutrition reported inappropriate recommendations generated by ChatGPT, indicating that personalized dietary recommendations by ChatGPT involve unpredictable errors [32,39]. Therefore, LLMs should not be relied on to provide current nutritional advice without nutrition professionals [32,39].

Our results revealed further limitations pertaining to the negligence of criteria that are relevant for sleep education by the LLMs. In particular, the criterion “family commitments” did not exceed a Likert-scale median of 0 (IQR 0-0) across all LLMs and levels of input information granularities. Regarding the evaluation of all LLMs and input information granularities, “arousal the night before competition” attained a median of 0 (IQR 0-0 to 0-4) in 5 of 8 Likert-scale ratings, while “lifestyle” gained a maximum median of 3.5 (IQR 2.25-4), reflecting a neutral rating. These criteria were highlighted by recent research to influence both sleep quality and quantity [6,14]. Nastasi et al [40] reported similar results in assessing ChatGPT’s ability to provide appropriate responses to medical questions within care contexts. While the authors noted appropriate responses corresponding to clinical guidelines, they indicated insufficient recommendations in regard to personalized medical advice, especially neglecting social factors [40].

Collectively, our results reveal that tailored sleep recommendations generated by GPT-4o and Google Gemini exhibit deficiencies according to received Likert-scale ratings by experienced raters, particularly in neglecting relevant criteria for sleep education. Therefore, sleep recommendations by LLMs should be carefully reviewed by a qualified coach or sleep professional before being applied in sleep educational settings.

### Differences in Ratings of GPT-4o and Google Gemini

Our results demonstrate that sleep recommendations by GPT-4o generally were rated higher compared to those by Google Gemini. Of the 10 significant differences between the 2 LLMs in Likert-scale ratings, 9 favored GPT-4o (*P*=.001 to *P*=.04). Although the remaining scenarios (n=42) did not display significant results, our findings suggest a better quality of recommendations for sleep education by GPT-4o compared to Google Gemini.

Similar to our work, different authors compared different publicly available LLMs in different scenarios. For example, Günay et al [41] assessed GPT-4o and Google Gemini on 40 electrocardiogram cases and their responses to the most likely diagnosis. The results revealed that GPT-4o achieved higher accuracy compared to Gemini in electrocardiogram diagnostics. Carlà et al [42] evaluated ChatGPT and Google Gemini on 4 retinal detachment cases related to planned surgeries and revealed that ChatGPT received higher ratings for accuracy and precision compared to Gemini. Hieronimus et al [43] analyzed the completeness and accuracy of dietary reference intake in meal plans for different dietary patterns created by ChatGPT and Google Bard (subsequently rebranded Gemini) and revealed higher quality in the response of ChatGPT. In hand surgery, it was shown that Google Gemini outperformed ChatGPT in classifying injuries, while ChatGPT provided more sensitive recommendations regarding surgical interventions [44].

Collectively, it appears that different LLMs differ in output quality, irrespective of the use case, and there seems to be a tendency that versions of ChatGPT outperform Google Gemini (which is in line with the results of our study), even though such statements need further investigation.

### Differences in Quality Regarding Prompt Information Granularity

Our results indicate that higher input granularity leads to better-rated sleep recommendations, independent of the LLM. In the combined results of both use cases and LLMs, scenario 2 (higher input information granularity) received significantly higher Likert-scale ratings in 28 of 52 criteria items of relevance for sleep education (*P*=.001 to *P*=.049) compared to scenario 1 (low input information granularity), while scenario 1 attained higher Likert-scale ratings in 2 of 52 items (*P*=.001 to *P*=.009) compared to scenario 2.

Our finding is in line with research examining LLMs in other contexts [30,31,45-48]. For example, Kunze et al [48] inserted 20 knee complaints necessitating triage into ChatGPT-4 and investigated the accuracy and suitability rated by orthopedic sports medicine physicians. The authors stated that when providing additional input information, the accuracy of information output by ChatGPT-4 improved, particularly with enhancements in conservative management, surgical approaches, and related treatments [48].

In the context of endurance sports, Düking et al [30] provided 3 prompts with different levels of input information granularities to ChatGPT, resulting in 3 training plans aimed to improve running performance. Following an evaluation of these training plans by coaching experts, the authors indicated an increased quality with more input information provided [30].

Collectively, when using LLMs, it seems important for users to input a sufficient amount of information into LLMs to improve output quality, at least for the herein investigated scenario.

An additional point of interest is the quality and appropriateness of the scientific sources. Our results demonstrate that more input information leads to significantly higher ratings in items pertaining to the used scientific sources (*P*<.001), irrespective of the analyzed LLM. While higher input information granularity leads to improved sleep recommendations, the higher quality and appropriateness of the scientific sources may also contribute to these improvements. Since our analysis did not involve a differentiation between the effect of input information granularity and the use of scientific resources, future research should investigate both factors separately.

Conclusively, higher input information granularity leads to improved sleep recommendations and might be influenced by higher quality and appropriateness of the scientific sources. When supplying LLMs for sleep education, coaches and athletes should be highly aware of the appropriate input granularity and conduct a thorough review of the received output, potentially in consultation with an individual with strong experience in the field of sleep and athletic populations.

### Strengths, Limitations, and Future Research

Strengths of our study include the assessment of different publicly available LLMs (ie, GPT-4o and Google Gemini) in different use cases and with different input information granularity, allowing a detailed analysis of provided sleep recommendations to the athletic population. Another strength of our study is the involvement of experienced raters evaluating the recommendations of LLMs.

Our results are limited to GPT-4o and Google Gemini on the versions available on May 15, 2024. As LLMs show fast developments (eg, by being able to search the internet), transferring our results to newer versions should be performed with caution. Due to such fast developments, it appears necessary to develop assessment methods or frameworks to evaluate the quality of LLMs in different scenarios to inform practitioners of the quality of currently available LLMs. Additionally, our results are, strictly speaking, only valid for the herein tested prompts, and other prompts might yield different results.

Despite the fact that all raters were experienced or well-educated in the field of sleep, the interrater reliability of our study ranges between slight and fair agreements (0.183 to 0.296). The tendency toward low interrater reliability is in line with previous research [30]. It may indicate that no single approach is universally optimal or perfect regarding athlete sleep recommendations. This suggests that different experts might rate the recommendations provided by LLMs in the respective use case differently, for example, based on personal preferences or personal experiences. While our results hold practical insights for athletes seeking sleep recommendations from LLMs (eg, to insert detailed information), individual sleep coaches might disagree with the recommendations provided by LLMs.

It seems important to note that even though it was shown that sleep education approaches can result in enhanced sleep behavior [13,49-51], to the best of our knowledge, there is no scientific evidence available that sleep recommendations generated by LLMs improve aspects of sleep in the athletic population. Future studies should evaluate the effectiveness of sleep education provided by publicly available LLMs to improve aspects of sleep.

### Conclusions

Our study indicates that sleep recommendations generated by GPT-4o or Google Gemini are not rated optimally, independently of the level of input information granularity. However, our results demonstrate that GPT-4o provides better sleep recommendations to athletes compared to Google Gemini and that sleep recommendations improved with more detailed input information for both herein investigated LLMs. Collectively, for LLMs to be used in practice, it seems essential to insert detailed information into LLMs and to thoroughly review the provided sleep recommendations for athletic populations.

## Supplementary material

10.2196/71358 Multimedia Appendix 1 Conversation with ChatGPT4o.

10.2196/71358 Multimedia Appendix 2 Conversation with Google Gemini.

10.2196/71358 Multimedia Appendix 3 Results of the significance testing comparing training plans generated by Google Gemini and GPT-4o in response to different input information granularity.

## Abbreviations

GPT-4o: ChatGPT-4o
LLM: large language model
