# Supplementary material for: Assessment of Recommendations Provided to Athletes Regarding Sleep Education by GPT-4o and Google Gemini: Comparative Evaluation Study
Source: JMIR Form Res. 2025 Jul 8;9:e71358. doi: 10.2196/71358 (PMC12287982; doi:10.2196/71358)
Supplement: Multimedia Appendix 1 [file formative-v9-e71358-s001.docx]

**ChatGPT**

**Use Case 1; Scenario 1:**

*Input*

I feel tired in the morning and after waking up. I do not know why. This is especially worse close to soccer games. Can you give me advice on how to improve my sleep?

*ChatGPT Output*

Feeling tired in the morning and after waking up can be frustrating, especially when it affects your performance in activities like soccer games. Here are some tips to help you improve your sleep quality:

**1. Establish a Consistent Sleep Schedule**

Go to bed and wake up at the same time every day, even on weekends. This helps regulate your body’s internal clock.

**2. Create a Relaxing Bedtime Routine**

Wind down before bed by engaging in calming activities such as reading, listening to soothing music, or taking a warm bath.

Avoid screens (phones, tablets, computers) for at least an hour before bedtime as the blue light can interfere with your ability to fall asleep.

**3. Optimize Your Sleep Environment**

Ensure your bedroom is cool, quiet, and dark. Consider using blackout curtains, earplugs, or a white noise machine if needed.

Invest in a comfortable mattress and pillows. The right bedding can make a significant difference in sleep quality.

**4. Watch What You Eat and Drink**

Avoid large meals, caffeine, and alcohol before bedtime. Caffeine and nicotine are stimulants that can keep you awake, while alcohol can disrupt your sleep cycle.

Stay hydrated but try to limit fluids before bed to reduce nighttime awakenings to use the bathroom.

**5. Be Active During the Day**

Regular physical activity can help you fall asleep faster and enjoy deeper sleep. However, try to avoid vigorous exercise close to bedtime.

**6. Manage Stress and Anxiety**

Practice relaxation techniques such as deep breathing, meditation, or yoga to help manage stress and anxiety which can interfere with sleep.

Journaling before bed can also help clear your mind and reduce worries that might keep you awake.

**7. Evaluate Your Sleep Quality**

Track your sleep with a diary or a sleep app to identify patterns or issues that might be affecting your sleep.

Consider seeing a doctor if you have persistent trouble sleeping. Conditions like sleep apnea or insomnia may require professional intervention.

**8. Pre-Game Sleep Tips**

Avoid heavy meals and caffeine the night before a game.

Ensure adequate sleep leading up to the game, not just the night before. Accumulative sleep deprivation can affect your performance.

Nap wisely: If you need a nap on game day, keep it short (20-30 minutes) and avoid napping late in the day to not interfere with nighttime sleep.

Implementing these tips can help improve your sleep quality, making you feel more rested and ready for your soccer games. If problems persist, consulting with a healthcare provider or a sleep specialist may be beneficial.

**Use Case 1; Scenario 2**

*Input*

I am a 23 year old male highly trained/national level soccer player. I train 5 times a week and plus a game per week. I feel tired due to poor sleep at night since approximately a year. Especially the night before a game I sleep poorly. Can you give me advice on factors which might affect my sleep and how I can reduce the impact of these factors? Use only scientific literature and specifically, for each advice, state this literature and provide a reference list at the end

*ChatGPT Output*

Certainly! Here are several factors that might affect your sleep, along with strategies to improve your sleep quality, supported by scientific literature:

**Factors Affecting Sleep and Solutions**

**Anxiety and Pre-Game Nerves:**

Impact: Anxiety about performance can disrupt sleep, particularly the night before a game.

Solution: Cognitive-behavioral strategies, such as relaxation techniques and cognitive restructuring, can help manage pre-game anxiety.

Literature:

Harvey, A. G., & Tang, N. K. Y. (2003). Cognitive behaviour therapy for primary insomnia: Can we rest yet? Sleep Medicine Reviews, 7(3), 237-262.

**Training Load:**

Impact: Intense physical training, especially close to bedtime, can elevate adrenaline levels and delay sleep onset.

Solution: Schedule intense training sessions earlier in the day and incorporate adequate recovery protocols.

Literature:

Halson, S. L. (2014). Monitoring training load to understand fatigue in athletes. Sports Medicine, 44(2), 139-147.

**Nutrition and Hydration:**

Impact: Poor nutrition and hydration habits can affect sleep quality. For example, caffeine and heavy meals close to bedtime can disrupt sleep.

Solution: Avoid caffeine in the late afternoon and evening. Opt for lighter meals in the evening.

Literature:

Grandner, M. A., & Kripke, D. F. (2004). Self-reported sleep complaints with long and short sleep: A nationally representative sample. Psychosomatic Medicine, 66(2), 239-241.

**Electronic Devices:**

Impact: Exposure to blue light from electronic devices can suppress melatonin production, making it harder to fall asleep.

Solution: Limit screen time at least one hour before bed and use blue light filters on devices if necessary.

Literature:

Chang, A. M., Aeschbach, D., Duffy, J. F., & Czeisler, C. A. (2015). Evening use of light-emitting eReaders negatively affects sleep, circadian timing, and next-morning alertness. Proceedings of the National Academy of Sciences, 112(4), 1232-1237.

**Sleep Environment:**

Impact: An uncomfortable sleep environment (e.g., too hot, noisy, or bright) can disrupt sleep.

Solution: Ensure your sleep environment is cool, quiet, and dark. Consider using earplugs, eye masks, or white noise machines if necessary.

Literature:

Stepanski, E. J., & Wyatt, J. K. (2003). Use of sleep hygiene in the treatment of insomnia. Sleep Medicine Reviews, 7(3), 215-225.

**Sleep Schedule and Routine:**

Impact: Irregular sleep patterns can disrupt your body's circadian rhythm, making it harder to fall asleep and wake up naturally.

Solution: Maintain a consistent sleep schedule, even on weekends. Develop a pre-sleep routine to signal to your body that it is time to wind down.

Literature:

Monk, T. H., Buysse, D. J., Rose, L. R., Hall, J. A., & Kupfer, D. J. (2000). The sleep of healthy people—a diary study. Chronobiology International, 17(1), 49-60.

References

Harvey, A. G., & Tang, N. K. Y. (2003). Cognitive behaviour therapy for primary insomnia: Can we rest yet? Sleep Medicine Reviews, 7(3), 237-262.

Halson, S. L. (2014). Monitoring training load to understand fatigue in athletes. Sports Medicine, 44(2), 139-147.

Grandner, M. A., & Kripke, D. F. (2004). Self-reported sleep complaints with long and short sleep: A nationally representative sample. Psychosomatic Medicine, 66(2), 239-241.

Chang, A. M., Aeschbach, D., Duffy, J. F., & Czeisler, C. A. (2015). Evening use of light-emitting eReaders negatively affects sleep, circadian timing, and next-morning alertness. Proceedings of the National Academy of Sciences, 112(4), 1232-1237.

Stepanski, E. J., & Wyatt, J. K. (2003). Use of sleep hygiene in the treatment of insomnia. Sleep Medicine Reviews, 7(3), 215-225.

Monk, T. H., Buysse, D. J., Rose, L. R., Hall, J. A., & Kupfer, D. J. (2000). The sleep of healthy people—a diary study. Chronobiology International, 17(1), 49-60.

Implementing these strategies should help improve your sleep quality and overall performance.

**Use Case 2; Scenario 1**

*Input*

I feel tired in the morning and after waking up. I do not know why. Maybe it is due to my running training or due to entering master level at university. Can you give me advice on how to improve my sleep?

*ChatGPT Output*

Feeling tired in the morning and after waking up can be influenced by several factors, including physical activity, academic stress, and sleep quality. Here are some tips to improve your sleep:

**1. Establish a Consistent Sleep Schedule**

Go to bed and wake up at the same time every day, even on weekends. This helps regulate your body's internal clock.

**2. Create a Relaxing Bedtime Routine**

Wind down before bed with relaxing activities like reading, meditating, or taking a warm bath.

Avoid screens (phones, computers, TVs) at least an hour before bed, as the blue light can interfere with melatonin production.

**3. Optimize Your Sleep Environment**

Keep your bedroom cool, dark, and quiet.

Invest in a comfortable mattress and pillows.

Use blackout curtains or an eye mask to block out light and earplugs or a white noise machine to mask noise.

**4. Watch Your Diet and Exercise**

Avoid large meals, caffeine, and alcohol close to bedtime. These can disrupt sleep.

Stay hydrated, but try to drink most of your fluids earlier in the day to avoid waking up to use the bathroom.

Exercise regularly but try to finish intense workouts at least a few hours before bed. Running can be stimulating, so ensure you have adequate time to wind down afterward.

**5. Manage Stress and Anxiety**

Practice stress-reducing techniques such as mindfulness, deep breathing exercises, or yoga.

Consider writing down your thoughts or a to-do list before bed to clear your mind.

**6. Monitor Your Sleep**

Use a sleep tracker or a sleep diary to understand your sleep patterns and identify potential issues.

**7. Limit Naps**

If you need to nap, keep it short (20-30 minutes) and avoid napping late in the day.

**8. Consider External Factors**

Review your running training program: Intense training can impact sleep quality. Ensure you have rest days and don’t overtrain.

Academic stress: As you enter the master’s level, stress can increase. Time management and stress-relief activities can help.

**9. Seek Professional Help if Needed**

If you consistently struggle with sleep despite trying these tips, consider consulting a doctor or a sleep specialist. There might be underlying issues such as sleep apnea, insomnia, or other sleep disorders.

Improving sleep quality can take time, so be patient with yourself as you implement these changes. Consistency is key to making lasting improvements.

**Use Case 2, Scenario 2:**

*Input*

I am a 25 year old female male tier 3, highly trained/national level marathon runner player and I run approx. 100km per week with an pyramidal intensity distribution. I feel tired in the morning since approximately entering my master course at university. Can you give me advice on factors which might affect my sleep and how I can reduce the impact of these factors? Use only scientific literature and specifically, for each advice, state this literature and provide a reference list at the end

*ChatGPT Output*

To address the factors that might be affecting your sleep and how to reduce their impact, here are some scientifically backed insights and strategies:

**1. Training Load and Timing**

Impact: High training volumes and intense exercise, especially close to bedtime, can impact sleep quality and duration.

Advice: Try to schedule high-intensity workouts earlier in the day. Research shows that evening exercise can disrupt sleep due to increased core body temperature and delayed melatonin production.

Reference: Oda, S., & Shirakawa, K. (2014). Sleep onset is disrupted following pre-sleep exercise that causes large physiological excitement at bedtime. European Journal of Applied Physiology, 114(9), 1789-1799.

**2. Academic Stress**

Impact: Academic stress can lead to increased anxiety and disrupted sleep patterns.

Advice: Implement stress management techniques such as mindfulness, meditation, or yoga. These practices have been shown to reduce stress and improve sleep quality.

Reference: Hershner, S. D., & Chervin, R. D. (2014). Causes and consequences of sleepiness among college students. Nature and Science of Sleep, 6, 73-84.

**3. Sleep Hygiene**

Impact: Poor sleep hygiene, such as irregular sleep schedules, exposure to screens before bedtime, and an unsuitable sleep environment, can affect sleep quality.

Advice: Establish a regular sleep schedule, limit screen time before bed, and create a sleep-conducive environment (cool, dark, and quiet).

Reference: Irish, L. A., Kline, C. E., Gunn, H. E., Buysse, D. J., & Hall, M. H. (2015). The role of sleep hygiene in promoting public health: A review of empirical evidence. Sleep Medicine Reviews, 22, 23-36.

**4. Nutrition and Hydration**

Impact: Poor nutritional habits and dehydration can interfere with sleep quality.

Advice: Ensure a balanced diet rich in nutrients and avoid heavy meals, caffeine, and alcohol close to bedtime. Staying hydrated throughout the day is also important but try to limit fluid intake in the evening to reduce nocturnal awakenings.

Reference: St-Onge, M. P., Mikic, A., & Pietrolungo, C. E. (2016). Effects of diet on sleep quality. Advances in Nutrition, 7(5), 938-949.

**5. Overtraining Syndrome**

Impact: Overtraining can lead to chronic fatigue, mood disturbances, and sleep problems.

Advice: Monitor your training load and ensure you incorporate adequate rest and recovery periods. Listening to your body and adjusting your training intensity accordingly is crucial to prevent overtraining.

Reference: Kellmann, M., & Kallus, K. W. (2001). Recovery-stress questionnaire for athletes: User manual. Human Kinetics.

**6. Light Exposure**

Impact: Exposure to natural light during the day and minimizing artificial light at night can regulate your circadian rhythms and improve sleep.

Advice: Increase exposure to natural daylight, especially in the morning, and minimize exposure to blue light from screens in the evening.

Reference: Cajochen, C. (2007). Alerting effects of light. Sleep Medicine Reviews, 11(6), 453-464.

**7. Sleep Environment**

Impact: The sleep environment plays a critical role in sleep quality.

Advice: Optimize your sleep environment by ensuring a comfortable mattress and pillows, and maintain a cool room temperature. Consider using blackout curtains and white noise machines if necessary.

Reference: Hirshkowitz, M., Whiton, K., Albert, S. M., Alessi, C., Bruni, O., DonCarlos, L., ... & Adams Hillard, P. J. (2015). National Sleep Foundation’s sleep time duration recommendations: methodology and results summary. Sleep Health, 1(1), 40-43.

**Reference List**

Oda, S., & Shirakawa, K. (2014). Sleep onset is disrupted following pre-sleep exercise that causes large physiological excitement at bedtime. European Journal of Applied Physiology, 114(9), 1789-1799.

Hershner, S. D., & Chervin, R. D. (2014). Causes and consequences of sleepiness among college students. Nature and Science of Sleep, 6, 73-84.

Irish, L. A., Kline, C. E., Gunn, H. E., Buysse, D. J., & Hall, M. H. (2015). The role of sleep hygiene in promoting public health: A review of empirical evidence. Sleep Medicine Reviews, 22, 23-36.

St-Onge, M. P., Mikic, A., & Pietrolungo, C. E. (2016). Effects of diet on sleep quality. Advances in Nutrition, 7(5), 938-949.

Kellmann, M., & Kallus, K. W. (2001). Recovery-stress questionnaire for athletes: User manual. Human Kinetics.

Cajochen, C. (2007). Alerting effects of light. Sleep Medicine Reviews, 11(6), 453-464.

Hirshkowitz, M., Whiton, K., Albert, S. M., Alessi, C., Bruni, O., DonCarlos, L., ... & Adams Hillard, P. J. (2015). National Sleep Foundation’s sleep time duration recommendations: methodology and results summary. Sleep Health, 1(1), 40-43.

By addressing these factors, you can potentially improve your sleep quality and overall recovery, which are critical for both your academic and athletic performance.
