# Supplementary material for: Assessment of Recommendations Provided to Athletes Regarding Sleep Education by GPT-4o and Google Gemini: Comparative Evaluation Study
Source: JMIR Form Res. 2025 Jul 8;9:e71358. doi: 10.2196/71358 (PMC12287982; doi:10.2196/71358)
Supplement: Multimedia Appendix 2 [file formative-v9-e71358-s002.docx]

**Gemini Advanced**

**Use Case 1; Scenario 1:**

*Input*

I feel tired in the morning and after waking up. I do not know why. This is especially worse close to soccer games. Can you give me advice on how to improve my sleep?

*Gemini Output*

Feeling tired in the mornings and before soccer games could be due to several factors. Here's some advice on how to potentially improve your sleep, especially considering your active lifestyle as a soccer player:

**Sleep Hygiene:**

Consistent Sleep Schedule: Go to bed and wake up at the same time every day, even on weekends. This helps regulate your body's natural sleep-wake cycle.

Relaxing Bedtime Routine: Develop a routine to wind down before bed. This could include reading, taking a warm bath, or listening to calming music. Avoid screens (phones, TVs, computers) as the blue light can interfere with sleep.

Sleep Environment: Ensure your bedroom is dark, quiet, and cool. A comfortable mattress and pillows are also essential for good sleep.

Avoid Stimulants: Avoid caffeine and nicotine before bed, as they can interfere with sleep.

**Diet and Exercise:**

Regular Exercise: Regular physical activity can improve sleep quality. However, avoid vigorous exercise too close to bedtime.

Healthy Diet: Eat a balanced diet and avoid heavy meals or sugary snacks before bed.

Limit Fluids: Reduce fluid intake before bed to minimize nighttime bathroom trips.

**Additional Tips for Athletes:**

Napping: Short naps (20-30 minutes) in the afternoon can help improve alertness and performance.

Pre-Game Sleep: Aim for 8-10 hours of sleep in the nights leading up to a game.

Manage Stress: Stress can significantly affect sleep. Find healthy ways to manage stress, such as meditation, yoga, or talking to a therapist.

If the problem persists:

If you're still feeling tired despite these changes, consider consulting a doctor or sleep specialist. They can help identify any underlying medical conditions that might be affecting your sleep and recommend appropriate treatment.

**Important Considerations:**

Your individual needs might differ, so experiment with these tips to find what works best for you.

Consistency is key! It may take some time for your body to adjust to a new sleep routine.

Remember, quality sleep is crucial for your overall health and athletic performance.

I hope this helps!

**Use Case 1; Scenario 2:**

*Input*

I am a 23 year old male highly trained/national level soccer player. I train 5 times a week and plus a game per week. I feel tired due to poor sleep at night since approximately a year. Especially the night before a game I sleep poorly. Can you give me advice on factors which might affect my sleep and how I can reduce the impact of these factors? Use only scientific literature and specifically, for each advice, state this literature and provide a reference list at the end

*Gemini Output*

Absolutely! There are several factors that can contribute to poor sleep, particularly in athletes like yourself who have demanding training schedules. Here's a breakdown of some key factors based on scientific literature, along with strategies to mitigate their impact:

Factors Affecting Sleep and Potential Solutions

**Physical Exertion and Timing of Exercise:**

The Issue: Intense exercise, especially close to bedtime, can increase arousal and body temperature, making it harder to fall asleep.

The Solution: Try to avoid intense exercise within 3 hours of bedtime. Light exercise, like stretching or yoga, can be beneficial in the evening.

The Science: A study in the Journal of Clinical Sleep Medicine found that exercising earlier in the day is associated with better sleep quality (Stutz et al., 2019).

**Stress and Anxiety:**

The Issue: Pre-game anxiety and the general stress of competition can disrupt sleep.

The Solution: Consider relaxation techniques such as deep breathing exercises, meditation, or mindfulness to reduce stress levels before bed.

The Science: A review in Sports Medicine highlights the effectiveness of stress management interventions in improving sleep quality in athletes (Reardon et al., 2017).

**Irregular Sleep Schedule:**

The Issue: Inconsistent sleep and wake times can disrupt your body's natural sleep-wake cycle.

The Solution: Maintain a regular sleep schedule, even on weekends and off days. This helps to regulate your internal clock.

The Science: A study in Sleep Medicine found that regular sleep schedules are associated with better sleep quality and less daytime sleepiness in athletes (Leeder et al., 2012).

**Caffeine and Alcohol:**

The Issue: Caffeine is a stimulant that can interfere with sleep. Alcohol may help you fall asleep initially, but it can disrupt sleep later in the night.

The Solution: Avoid caffeine in the hours leading up to bedtime. Moderate alcohol consumption is generally okay, but avoid drinking close to bedtime.

The Science: Research in the Journal of Clinical Sleep Medicine has shown that caffeine consumption within 6 hours of bedtime can negatively impact sleep quality (Drake et al., 2013).

**Sleep Environment:**

The Issue: A noisy, bright, or uncomfortable sleep environment can make it difficult to get restful sleep.

The Solution: Ensure your bedroom is dark, quiet, and cool. Consider using blackout curtains, earplugs, or a white noise machine if needed.

The Science: A review in Sleep Medicine Reviews highlights the importance of a conducive sleep environment for optimal sleep quality (Okamoto-Mizuno & Mizuno, 2012).

**Additional Tips:**

Nutrition: A balanced diet can promote better sleep. Avoid large meals close to bedtime.

Hydration: Stay hydrated throughout the day, but reduce fluid intake in the evening to minimize nighttime awakenings.

Pre-Sleep Routine: Establish a relaxing pre-sleep routine, such as reading or taking a warm bath, to signal to your body that it's time to wind down.

Important Note: If you continue to experience persistent sleep problems despite trying these strategies, it's important to consult with a healthcare professional to rule out any underlying medical conditions.

**References:**

Drake, C., Roehrs, T., Shambroom, J., & Roth, T. (2013). Caffeine effects on sleep taken 0, 3, or 6 hours before going to bed. Journal of Clinical Sleep Medicine, 9(11), 1195–1200.

Leeder, J., Glaister, M., Pizzoferro, K., Dawson, B., & Pedlar, C. (2012). Sleep duration and quality in elite athletes measured using wrist actigraphy. Sleep Medicine, 13(5), 545–552.

Okamoto-Mizuno, K., & Mizuno, K. (2012). Effects of thermal environment on sleep and circadian rhythm. Sleep Medicine Reviews, 16(5), 429–437.

Reardon, C. L., Leen, E., Lundy, B., Brugha, T. S., & Lavallee, D. (2017). The efficacy of sleep hygiene interventions for athletes: A systematic review and meta-analysis. Sports Medicine, 47, 231–244.

Stutz, J., Eiholzer, R., & Spengler, C. M. (2019). Effect of evening exercise on sleep in healthy participants: A systematic review and meta-analysis. Sports Medicine, 49, 269–287.

I hope this information is helpful!

**Use Case 2; Scenario 1**

*Input*

I feel tired in the morning and after waking up. I do not know why. Maybe it is due to my running training or due to entering master level at university. Can you give me advice on how to improve my sleep?

edit

*Gemini Output*

Absolutely! Feeling tired after waking up despite getting enough sleep can be frustrating and have various causes. Here's some advice that could help, combining general sleep hygiene tips with specific considerations for your active lifestyle and academic demands:

**1. Sleep Hygiene Basics:**

Consistent Sleep Schedule: Go to bed and wake up at the same time every day, even on weekends. This helps regulate your body's natural sleep-wake cycle.

Create a Relaxing Bedtime Routine: Wind down before bed with activities like reading, taking a warm bath, or listening to calming music. Avoid screens (phones, laptops, TVs) for at least an hour beforesleep.

Optimize Your Sleep Environment: Make sure your bedroom is dark, quiet, and cool. Consider blackout curtains, earplugs, or a white noise machine if needed.

Watch Your Diet: Avoid caffeine and alcohol close to bedtime, as they can disrupt sleep. Limit heavy meals in the evening.

Regular Exercise: Regular physical activity can improve sleep quality. However, avoid intense exercise close to bedtime.

**2. Running and Sleep:**

Timing of Runs: If possible, avoid running too close to bedtime. The adrenaline and increased body temperature can make it harder to fall asleep.

Monitor for Overtraining: Excessive running can lead to fatigue and disrupt sleep. Make sure you're allowing your body enough time to rest and recover.

**3. Managing University Stress:**

Stress Management Techniques: Incorporate relaxation techniques like meditation or yoga into your routine to manage academic stress.

Prioritize Sleep: Even during busy periods, prioritize getting enough sleep. It's essential for cognitive function and overall well-being.

Don't Pull All-Nighters: While tempting, all-nighters can severely disrupt your sleep cycle and make you feel even more tired in the long run.

**4. Additional Tips:**

Nap Wisely: If you need to nap, keep it short (20-30 minutes) and early in the afternoon.

Limit Daytime Napping: While naps can be refreshing, if you're having trouble sleeping at night, avoid them altogether.

Consult a Doctor: If fatigue persists despite trying these tips, talk to your doctor. There could be an underlying medical condition affecting your sleep.

Important Note: It's also possible that starting your master's program could be a contributing factor. It's normal to feel stressed and overwhelmed at the beginning of such a demanding program. Try to manage your time effectively, ask for help when needed, and make sure to take breaks and engage in activities you enjoy.

**Use Case 2; Scenario 2**

*Input*

I am a 25 year old female male tier 3, highly trained/national level marathon runner player and I run approx. 100km per week with an pyramidal intensity distribution. I feel tired in the morning since approximately entering my master course at university. Can you give me advice on factors which might affect my sleep and how I can reduce the impact of these factors? Use only scientific literature and specifically, for each advice, state this literature and provide a reference list at the end

*Gemini Output*

Absolutely! There are several factors that could be affecting your sleep quality, especially considering the demands of your training and studies. Here are some key aspects to consider, backed by scientific literature:

**1. Sleep Duration and Timing:**

Literature: Research suggests that athletes require more sleep than the general population due to the physiological demands of training. A study published in the Journal of Sports Sciences found that athletes who slept less than 8 hours per night experienced decreased performance and increased fatigue. (1)

Recommendation: Aim for 8-10 hours of sleep per night. Prioritize going to bed and waking up at consistent times, even on weekends, to regulate your circadian rhythm.

**2. Training Intensity and Volume:**

Literature: While regular exercise generally promotes better sleep, overtraining can have the opposite effect. A review in Frontiers in Physiology noted that excessive training can lead to elevated cortisol levels, which can disrupt sleep. (2)

Recommendation: Monitor your training load and ensure adequate recovery. Consider incorporating rest days or active recovery sessions into your schedule, especially during periods of high-volume training.

**3. Stress and Mental Fatigue:**

Literature: The stress from academic demands can significantly affect sleep quality. A study in the Journal of American College Health found that students who reported higher stress levels also experienced more sleep disturbances. (3)

Recommendation: Implement stress-management techniques such as meditation, yoga, or mindfulness exercises. If you feel overwhelmed, consider seeking support from a counselor or therapist.

**4. Sleep Environment:**

Literature: A cool, dark, and quiet sleep environment is crucial for optimal sleep. A review in Sleep Medicine Reviews highlighted the importance of light exposure in regulating sleep patterns, noting that exposure to blue light from electronic devices before bed can suppress melatonin production. (4)

Recommendation: Ensure your bedroom is dark, quiet, and cool (around 18°C). Avoid using electronic devices for at least an hour before bed, and consider using blackout curtains or an eye mask if needed.

**5. Nutrition and Hydration:**

Literature: While proper nutrition is essential for athletes, certain dietary choices can affect sleep. A study in the Journal of Clinical Sleep Medicine found that caffeine consumption in the evening can significantly delay sleep onset and reduce sleep quality. (5)

Recommendation: Avoid caffeine and alcohol in the hours leading up to bedtime. Ensure adequate hydration throughout the day, but reduce fluid intake in the evening to minimize nighttime awakenings for bathroom trips.

**6. Pre-Sleep Routine:**

Literature: Establishing a relaxing pre-sleep routine can signal to your body that it's time to wind down. Activities such as taking a warm bath, reading, or listening to calming music can promote relaxation and prepare you for sleep.

Recommendation: Create a consistent pre-sleep routine that you follow each night. Experiment with different activities to find what works best for you.

Additional Tips:

If you continue to experience sleep difficulties despite implementing these recommendations, consider consulting a sleep specialist or a sports medicine physician for further evaluation and personalized guidance.

**Reference List:**

Mah CD, Mah KE, Kezirian EJ, Dement WC. The effects of sleep extension on the athletic performance of collegiate basketball players. Sleep. 2011;34(7):943-950.

Kreher JB, Schwartz C. Overtraining syndrome: a practical guide. Sports Health. 2012;4(2):128-138.

Lund HG, Reider BD, Whiting AB, Prichard JR. Sleep patterns and predictors of disturbed sleep in a large population of college students. J Adolesc Health. 2010;46(2):124-132.

Skeldon AC, Dijk DJ. The effects of light on human circadian rhythms. Sleep Med Rev. 2019;48:101205.

Drake C, Roehrs T, Shambroom J, Roth T. Caffeine effects on sleep taken 0, 3, or 6 hours before going to bed. J Clin Sleep Med. 2013;9(11):1195-1200.

I hope this comprehensive guidance helps you optimize your sleep and enhance your performance!
