# Supplementary material for: Assessment of Recommendations Provided to Athletes Regarding Sleep Education by GPT-4o and Google Gemini: Comparative Evaluation Study
Source: JMIR Form Res. 2025 Jul 8;9:e71358. doi: 10.2196/71358 (PMC12287982; doi:10.2196/71358)
Supplement: Multimedia Appendix 3 [file formative-v9-e71358-s003.docx]

| **Relevant aspects when deploying sleep recommendations** | | **Significance testing (p-value) (different prompt, different LLM)** | | | |
| --- | --- | --- | --- | --- | --- |
|  | | Gem_C1-S1 vs GPT_C1-S2 | GPT_C1-S1 vs Gem_ C1-S2 | Gem_C2-S1 vs GPT_C2-S2 | GPT_C2-S1 vs Gem_ C2-S2 |
| **General Aspects** | Overall training plan | **0.003** | 0.407 | 0.378 | 0.300 |
|  | Training load | **< 0.001** | **< 0.001** | **< 0.001** | 0.342 |
|  | Unfamiliar sleeping environments | 0.121 | **0.021** | 0.075 | 0.256 |
|  | Early morning or late evening training or competition | **0.016** | **0.007** | 0.764 | 0.368 |
|  | Arousal the night before competition / training | **< 0.001** | 0.664 | **0.013** | 0.139 |
|  | Circadian rhythm disruptions / consistency of sleep schedule | **0.011** | 0.225 | 0.672 | 0.672 |
|  | Family commitments | 1.000 | **0.049** | 1.000 | 1.000 |
|  | Lifestyle choices | 0.354 | 0.192 | 0.623 | 0.157 |
|  | Use of electronic devices | **< 0.001** | **< 0.001** | 0.118 | 0.683 |
|  | Nutrition | **0.018** | 0.870 | 0.159 | 0.703 |
| **Scientific sources** | Is real and existing literature stated (no “fake citations”)? | **< 0.001** | **< 0.001** | **< 0.001** | **< 0.001** |
|  | Appropriateness of provided scientific evidence | **< 0.001** | **< 0.001** | **< 0.001** | **< 0.001** |
|  | Quality of provided scientific evidence in this specific context | **< 0.001** | **< 0.001** | **< 0.001** | **< 0.001** |

**Gem_C1-S1 = Google Gemini, Use Case 1 – Scenario 1; Gem_C1-S2 = Google Gemini, Use Case 1 – Scenario 2; Gem_C2-S1 = Google Gemini, Use Case 2 – Scenario 1; Gem_C2-S2 = Google Gemini, Use Case 2 – Scenario 2; GPT_C1-S1 = Chat GPT, Use Case 1 – Scenario 1; GPT_C1-S2 = Chat GPT, Use Case 1 – Scenario 2; GPT_C2-S1 = Chat GPT, Use Case 2 – Scenario 1; GPT_C2-S2 = Chat GPT, Use Case 2 – Scenario 2.**
